# Rapidly progressive dementia: criteria proposal

**DOI:** 10.1002/alz.088025

**Published:** 2025-01-09

**Authors:** Nihal Satyadev, Philip W Tipton, S Richard Dunham, Matthew R Brier, John C. Morris, Neill R Graff‐Radford, Gregory S Day

**Affiliations:** ^1^ Mayo Clinic, Jacksonville, FL USA; ^2^ Washington University School of Medicine, Saint Louis, MO USA; ^3^ Washington University in St. Louis, School of Medicine, St. Louis, MO USA; ^4^ Department of Neurology, Mayo Clinic, Jacksonville, FL USA

## Abstract

**Background:**

Rapidly progressive dementia (RPD) is variably defined across published cohorts. A standardized definition is needed to support multicenter studies required to inform the causes of RPD and optimize recognition and management of treatment‐responsive causes. An optimal definition will capture the broad spectrum of causes of RPD, adequately differentiate patients with rapid and typically progressive presentations of neurodegenerative disease, and be reliably implemented across healthcare settings and centers.

**Method:**

The Clinical Dementia Rating® (CDR) is a validated dementia staging tool that is widely used in clinical trials and observational studies. We applied the CDR to diagnose RPD in patients who developed dementia (CDR ≥1) within 1 year or incapacitation (CDR ≥2) within 2 years of symptom onset. This definition was prospectively applied to patients with suspected RPD at Mayo Clinic in Florida (MCF: Jacksonville, FL; Jan 2020 – Oct 2023) and Washington University in St. Louis (WU: Saint Louis, MO; Jun 2016 – Dec 2019), and retrospectively applied to individuals within the National Alzheimer’s Coordinating Center (NACC) dataset. Criteria performance was compared across cohorts.

**Result:**

Two‐hundred twenty‐six patients were referred with suspected RPD at MCF‐WU. Of these, 155/226 (68.6%) met our proposed RPD criteria, including patients with Alzheimer disease and related dementias (ADRD; 34/155, 21.9%), Creutzfeldt Jacob disease (CJD; 25/155, 16.1%), autoimmune encephalitis (52/155, 33.5%), vascular pathologies (14/155, 9.0%), toxic/metabolic disruptions (7/155, 4.5%) and primary psychiatric disorders (4/155, 2.6%). A small subset of NACC participants met our proposed RPD criteria (836/20,418; 0.04%), emphasizing the unique nature of RPD. Causes of RPD in NACC included ADRD (676/836, 80.1%), CJD (72/836, 8.6%), and vascular pathologies (24/836, 2.9%). Rates of progression (∆CDR sum‐of‐boxes/year) clearly distinguished patients with RPD from non‐RPD in both cohorts (MCF‐WU, 13.6±5.8 vs 4.7±5.9, p<0.001; NACC, 6.7±3.1 vs. 1.7±1.4, p<0.001).

**Conclusion:**

Our CDR‐based definition of RPD distinguished patients with a broad spectrum of causes of RPD from those with typically progressive dementia across multiple cohorts. Broad application of this criteria would standardize diagnosis of RPD and support the design and implementation of multicenter studies RPD.

